# Predictive factors for failure of the levonorgestrel releasing intrauterine system in women with heavy menstrual bleeding

**DOI:** 10.1186/s12905-021-01210-x

**Published:** 2021-02-09

**Authors:** Pleun Beelen, Marian J. van den Brink, Malou C. Herman, Peggy M. Geomini, Ruben G. Duijnhoven, Marlies Y. Bongers

**Affiliations:** 1 Department of Obstetrics and Gynecology, Máxima MC, 5504 DB Veldhoven, The Netherlands; 2 Department of General Practice, University of Maastricht, Maastricht, The Netherlands; 3 Department of General Practice and Elderly Care Medicine, University of Groningen, University Medical Centre Groningen, Groningen, The Netherlands; 4 Department of Obstetrics and Gynecology, Jeroen Bosch Hospital, ‘s Hertogenbosch, The Netherlands; 5 Department of Obstetrics and Gynecology, Academic Medical Centre, Amsterdam, The Netherlands; 6 Research School Grow, University of Maastricht, Maastricht, The Netherlands

**Keywords:** Heavy menstrual bleeding, Levonorgestrel intrauterine system, Failure, Prognosis

## Abstract

**Background:**

This study was conducted to identify factors that are associated with failure of treatment using the levonorgestrel releasing intrauterine system (LNG-IUS) in women with heavy menstrual bleeding.

**Methods:**

For this study, data of a cohort of women treated with an LNG-IUS was used. Women who suffered from heavy menstrual bleeding, aged 34 years and older, without intracavitary pathology and without a future child wish, were recruited in hospitals and general practices in the Netherlands. Eight potential prognostic baseline variables (age, body mass index, caesarean section, vaginal delivery, previous treatment, anticoagulant use, dysmenorrhea, and pictorial blood assessment score) were analyzed using univariable and multivariable regression models to estimate the risk of failure. The main outcome measure was discontinuation of the LNG-IUS within 24 months of follow up, defined as removal of the LNG-IUS or receiving an additional intervention.

**Results:**

A total of 209 women received the LNG-IUS, 201 women were included in the analyses. 93 women (46%) discontinued LNG-IUS treatment within 24 months. Multivariable analysis showed younger age (age below 45) (adjusted RR 1.51, 95% CI 1.10–2.09, p = .012) and severe dysmenorrhea (adjusted RR 1.36, 95% CI 1.01–1.82, p = .041) to be associated with a higher risk of discontinuation.

**Conclusions:**

High discontinuation rates are found in women who receive an LNG-IUS to treat heavy menstrual bleeding. A younger age and severe dysmenorrhea are found to be risk factors for discontinuation of LNG-IUS treatment. These results are relevant for counselling women with heavy menstrual bleeding.

## Background

The levonorgestrel releasing intrauterine system (LNG-IUS) is frequently used in women of reproductive age. It has both contraceptive as well as non-contraceptive purposes, especially as treatment for heavy menstrual bleeding (HMB) [1–3]. Up to one third of women of reproductive age experience HMB which can be disruptive to their daily life [4, 5]. The LNG-IUS is known to be effective in reducing menstrual bleeding and increasing quality of life [6, 7]. It has several favorable characteristics compared with other more invasive treatment options like endometrial ablation and hysterectomy. Most importantly, it provides reversible treatment, while it also has a contraceptive effect and it can be inserted by the general practitioner. Despite these benefits and its effectiveness, about 40% of women discontinue LNG-IUS treatment, mostly because of persistent heavy bleeding or side effects such as irregular bleeding [6–8].

To identify women who are at higher risk of treatment failure, it is important to know which factors predict this failure. Few studies investigated prognostic factors of LNG-IUS treatment in women with HMB [6, 9–13]. In these studies, five prognostic factors were described to be associated with failure: BMI, amount of blood loss, myomas, dysmenorrhea and depression. The results of these studies, however, were inconsistent. By conducting this study, we aim to add to the existing evidence and to identify possible factors associated with failure of the LNG-IUS in women with HMB. With the results counselling might be tailored to the individual woman and discontinuation rates might be reduced.

## Methods

Data were collected from women with HMB, participating in the MIRA-trial, or its observational cohort [7, 14]. The MIRA-trial was conducted according to the principles of the Declaration of Helsinki and approved by the ethics committee of the Academic Medical Centre Amsterdam, the Netherlands (Registration Number 2011-372). In the MIRA-trial the effectiveness of the LNG-IUS 52 mg (Mirena®, Bayer HealthCare Pharmaceuticals, Germany) was compared with bipolar radiofrequency endometrial ablation (NovaSure®, Hologic, Marlborough, MA, USA) for the treatment of HMB. Women who declined participation in the trial were asked to participate in the observational cohort for follow-up. Women aged 34 years or older, with no future pregnancy wish were included in the study. Women were excluded if they had abnormal cervix cytology in the past five years, a uterus length of more than ten centimeters, intracavitary fibroids or polyps or large intramural fibroids (≥ 3 cm) determined by either bimanual vaginal examination or a transvaginal ultrasound depending on the setting (general practice or gynecology department).

Data collection was done for all women equally, before start of treatment and at 3, 6, 12, and 24 months. Women were asked to fill in questionnaires about potential reinterventions and their reasons. Additionally, research nurses of the participating centers collected reintervention data from medical records.

Full details of the protocol have been published previously [14]. Women had been recruited between 2012 and 2016 in 26 hospitals and in a network of general practices in the Netherlands. For the present study we used data on outcomes of women who were treated with LNG-IUS in either the trial or cohort. The objective of this study was to identify prognostic factors which are associated with failure. Failure was defined as discontinuation of the LNG-IUS, meaning removal of the LNG-IUS or receiving an additional intervention within the follow-up period of 24 months.

The following characteristics were collected at baseline: age, body mass index (BMI, calculated as weight in kilograms divided by height in meters squared), number of vaginal deliveries, caesarean section, tubal ligation, previous LNG-IUS, previous uterine surgery, previous HMB treatment, anticoagulant use, dysmenorrhea, duration of menstruation (days) and pictorial blood assessment chart (PBAC)-score. Data of discontinuation, the type of reinterventions (drug and surgical reinterventions) and the reason for reintervention were collected by questionnaires and from women’s medical records.

### Statistical analyses

The general rule of thumb studying one predictor variable for every 10 events was taken into account for the analysis. Eight potential prognostic factors were considered, based on previous research and on clinical knowledge: age, BMI, history of caesarean section, vaginal delivery, previous treatment for HMB, use of anticoagulants, dysmenorrhea, and baseline PBAC score [6, 9–13]. These characteristics were first tested in a univariable model. When fitting the model, the relation of these potential prognostic factors with failure of treatment was tested using various forms, including categorization, dichotomization or continuous. In the final model, all factors were included as dichotomized variables. Age was dichotomized as ≤ 45 or > 45 years, BMI as ≥ 25 or < 25 kg/m^2^, caesarean section, vaginal delivery, previous HMB treatment, and anticoagulant use as yes or no, and baseline PBAC score as ≥ 425 or < 425 (above and below baseline median PBAC score). Dysmenorrhea (measured with a 4-point Likert scale) was dichotomized as present or non-present, and to estimate the risk more specifically for women who experienced severe dysmenorrhea, we also dichotomized it as ‘severe’ or ‘none to moderate’. In addition, the model was adjusted for any effect of randomization by including a variable to code the RCT and observational data. All variables which were found to be risk factors in previous research together with statistically significant variables in the univariable analyses were entered in a multivariable logistic regression model using the forced entry method. Stochastic regression imputation was used to impute missing baseline data, with the assumption that values were missing at random. A sensitivity analysis was performed in which women who were included in general practice and did not receive an ultrasound were excluded, in order to rule out bias due to unknown intracavitary pathology.

Baseline continuous variables were presented either as means and standard deviations (SD), or median and interquartile range (IQR). Categorical data were presented as numbers and percentages. Relative risks (RR) with 95% confidence intervals (CI) were estimated using log-binomial regression. A significance level of 5% was used for all statistical tests. Statistical analyses were performed using the software Statistical Package for the Social Sciences (SPSS), version 25 (IBM Corp, New York, USA).

## Results

**Fig. 1:**
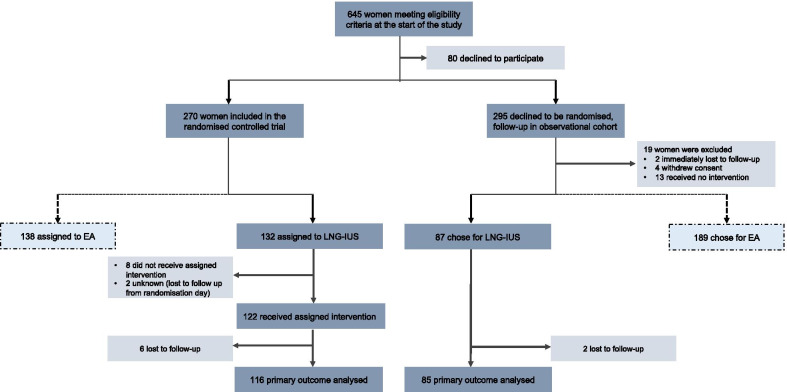
Flow chart. LNG-IUS, levonorgestrel releasing intrauterine system; EA, endometrial ablation

Between April 27, 2012 and January 12, 2016, 209 women received an LNG-IUS. One hundred twenty-two women were randomized and 87 women were followed in the observational cohort, eight women were lost to follow-up at 24 months of follow-up (Fig. 1). Data of 201 women were analyzed. In total 14 women (7.0%) were treated by the general practitioner; of which 10 (5.0%) women did not receive an ultrasound.

The mean age for women included in the study was 44.7 (SD 4.8), with a mean BMI of 27.4 (SD 5.6). Further baseline characteristics are provided in Table 1.

**Table 1:** Baseline characteristics

	LNG-IUS (N = 209)
Study arm	
*Cohort*	87 (42%)
*RCT*	122 (58%)
Age (mean ± SD)	44.7 ± 4.8
*Age* ≤ *45*	97 (46%)
*Age* > *45*	112 (54%)
BMI (mean ± SD)	27.4 ± 5.6
*BMI* < *25*	58 (39%)
*BMI* ≥ *25*	91 (61%)
Previous vaginal delivery	159 (81%)
Previous caesarean section	34 (17%)
Previous polypectomy/ myomectomy	13 (6.3%)
Tubal ligation	5 (2.4%)
Previous LNG-IUS^*^	19 (9.3%)
Previous treatment HMB^†^	100 (48%)
*Non-hormonal treatment*	33 (16%)
*Hormonal treatment*	81 (39%)
Anticoagulants	10 (4.8%)
Dysmenorrhea	139 (71%)
*Mild*	36 (18%)
*Moderate*	59 (30%)
*Severe*	44 (22%)
PBAC-score (median-IQR)	425 (288–744)
Bleeding days (median-IQR)	8 (6–11)

LNG-IUS, levonorgestrel releasing intrauterine system; BMI, Body Mass Index; HMB, heavy menstrual bleeding; PBAC, Pictorial Blood Assessment Chart. Data are presented as number of women (%) unless otherwise indicated

*Indication for treatment: contraception and/or HMB

^**†**^Women could have received ≥ one previous treatment

**Fig. 2:**
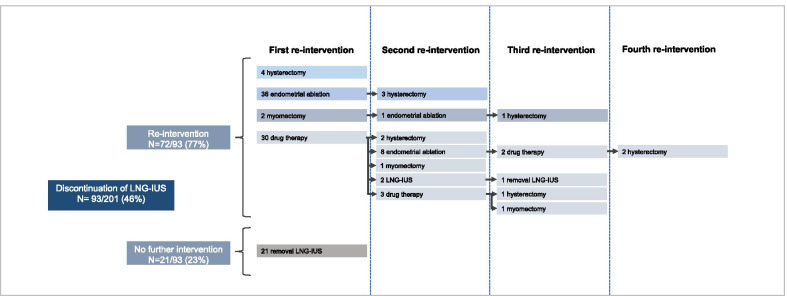
Treatment choice after failed treatment with the LNG-IUS. LNG-IUS, levonorgestrel releasing intrauterine system. Follow-up period of 24 months

In total 93/201 women (46%) discontinued treatment with the LNG-IUS. The most common reported reason for discontinuation was persistent HMB, which was reported in 42% of women. Of these women 56% (22/39) discontinued treatment within six months after LNG-IUS insertion, and 15% (6/39) discontinued treatment between one and two years after start. The same percentages were seen in the total group of women who discontinued treatment. Other frequently reported reasons were the presence of side effects (18%); most often mood symptoms, headache and weight gain, and the presence of dysmenorrhea or pelvic pain (17%). In Fig. 2 the treatment choice for each woman after failed LNG-IUS treatment is presented. 77% (72/93) of women who discontinued treatment received a reintervention. The reintervention most commonly performed was endometrial ablation (45/93, 48%). A hysterectomy was performed in 13 women (14%), of which 6 women had earlier undergone endometrial ablation. 30 women (32%) received (additional) drug therapy (tranexamic acid N = 13, combined oral contraceptive pill N = 11, LNG-IUS N = 5, progestogens N = 4, estrogens N = 2, antiprogestogens N = 2).

The results of the univariable analysis are presented in Table 2. Two factors were found to be associated with failure. Women aged 45 years old or below had a higher risk of failure compared to women above 45 years (RR 1.57, 95% CI 1.13- 2.16, P = 0.004). The presence of severe dysmenorrhea was also associated with a higher risk of failure compared to women with no to moderate dysmenorrhea (RR 1.44, 95% CI 1.06–1.94, P = 0.034). The multivariable analysis confirmed age and severe dysmenorrhea to be independently associated with failure of the LNG-IUS (adjusted RR for age ≤ 45 1.51, 95% CI 1.10–2.09, P = 0.012, adjusted RR for severe dysmenorrhea 1.36, 95% CI 1.01–1.82, P = 0.041) (Table 2). The most common reasons for discontinuation in both women of younger age and women with severe dysmenorrhea were persistent HMB and the presence of side effects. These reasons were similar to those of the total population.

**Table 2:** Univariate and multivariate analysis of predictors of failure of the LNG-IUS

Characteristics	Univariable analysis			Multivariable analysis		
	RR	95% CI	p-value	RR	95% CI	p-value
Study arm *(cohort vs RCT)*	1.02	0.76–1.39	.88	1.03	0.76–1.38	.87
Age *(*≤ *45 vs* > *45)*	**1.57**	**1.13–2.16**	**.004**	**1.51**	**1.10–2.09**	**.012**
BMI (≥ *25 vs* < *25)*	0.91	0.67–1.23	.54	0.97	0.72–1.31	.86
Caesarean section	1.36	0.97–1.89	.10			
Vaginal delivery	0.89	0.62–1.27	.49			
Previous treatment HMB	1.07	0.80–1.45	.64			
Anticoagulants	1.32	0.78–2.24	.52			
Dysmenorrhea						
Present versus non-present	1.04	0.75–1.44	0.83			
Severe versus none/mild/moderate	**1.44**	**1.06–1.94**	**.034**	**1.36**	**1.01–1.82**	**.041**
PBAC-score *(*≥ *425 vs* < *425)*	1.11	0.82–1.50	.49	1.08	0.81–1.43	.61

BMI, Body Mass Index; HMB, heavy menstrual bleeding; PBAC, Pictorial Blood Assessment Chart. Imputed data: BMI n = 60, caesarean section N = 3, previous treatment HMB N = 1, anticoagulants N = 1, dysmenorrhea N = 12, PBAC = 39. Multivariate analysis preceded by a forced entry method

The sensitivity analysis showed the same discontinuation rate; in total 46% (88/191) of women discontinued treatment with the LNG-IUS. Comparable results were found with the regression analyses, with age and severe dysmenorrhea both being significantly associated with failure (adjusted RR for age ≤ 45 1.52, 95% CI 1.10–2.11, P = 0.012, adjusted RR for severe dysmenorrhea 1.39, 95% CI 1.02–1.89, P = 0.035).

## Discussion

### Main findings

In this study we aimed to identify prognostic factors associated with failure of the LNG-IUS in women with HMB. Age below 45 years and the presence of severe dysmenorrhea were found to be independently associated with a higher risk of failure.

### Strengths and limitations

Strengths of this study are that the data was prospectively collected and the study had a relatively long follow-up period (24 months). Information about reinterventions was not only obtained from participants’ medical records, but also from patient questionnaires, with which we minimized the risk of missing data in women who did not receive a reintervention in the hospital where they received the LNG-IUS. The MIRA-trial was a pragmatic trial in which women could be treated at both the general practice as the gynecology department, reflecting daily practice. As a consequence, the underlying cause of HMB was not extensively examined and women who were included at the general practice did not routinely receive an ultrasound before insertion of the LNG-IUS. This is in line with the Dutch general practice guideline, in which this is described as an optional investigation [15]. One could argue that intracavitary pathology or a distorted uterine cavity by intramural fibroids might have been present in some of these women which could have led to a higher reintervention rate. Therefore, we performed a sensitivity analysis in which we excluded women where no ultrasound was performed (N = 10). The results of this sensitivity analysis were comparable with the results of the primary analysis. Another limitation of this study was the relatively small number of women included in the study, by which the number of potential prognostic factors included in the model was limited and some factors were possibly not found to be significant predictors due to a lack of power alone. To conduct a sample size estimation a priori*,* or power calculation *post-hoc* however was not feasible. Power in a prognostic model is driven by the number of prognostic factors in the model, the incidence of the outcome, and the number of observations [16]. A larger sample size can therefore always be considered preferable and a minimum required sample size is not straight forward. Simulation of different scenarios for sample size estimation as proposed by van Smeden et al. was not conducted [17].

For some baseline characteristics data was missing, most importantly BMI and PBAC-score. Multiple regression imputation was used to prevent a complete case analysis. Regression imputation is a commonly used and reliable method to impute missing data and preferred over complete case analysis. We combined the results of the RCT and the prospective cohort arm of the MIRA trial which caused methodological heterogeneity. Given, however, that both the RCT and observational data collection were conducted under the same protocol, we consider the effect of this heterogeneity small. Moreover, discontinuation rates between women randomized and women followed in the observational cohort were comparable and to rule out a small influence which we were not able to objectify, we added study arm to the multivariable analysis.

### Interpretation

Outcome predictors of LNG-IUS treatment in women with HMB are not extensively researched. Six studies were found which investigated potential prognostic factors [6, 9–13]. These studies used different outcome measures including quality of life, menstrual bleeding pattern, discontinuation of care and presence of HMB at follow-up or receiving a hysterectomy. Age was investigated as a prognostic factor for failure in three of these studies [6, 9, 12]. It was measured as a continuous factor or as dichotomous factor (≤ 35 or > 35 years) and was not found to be a risk factor for failure. A cut-off value of 45 was chosen in our study because this value was found to be associated with failure in endometrial ablation, another frequently applied HMB treatment, in previous research [18]. From a clinical perspective, the lower risk of failure of women who are at least 45 years old might be explained because these women approach (or already reached) menopause. Therefore, they might be more inclined to wait until their menstrual bleeding decreases spontaneously. Younger women have to bridge a longer period which could be the reason why they faster consider to remove the LNG-IUS and opt for another intervention.

Presence of dysmenorrhea in general was not shown to be associated with failure, though women with severe dysmenorrhea were found to have a higher failure risk. For clinical practice this implicates physicians should question women about the severity of their dysmenorrhea symptoms as this influences the chance of successful treatment. Two previous studies investigated if dysmenorrhea was associated with a higher failure risk. One study found no association [6] and the other study found abdominal and back pain to be related to the risk of receiving a hysterectomy [12]. In the present study the cause of dysmenorrhea was not investigated. Adenomyosis and endometriosis have been found to be important causes of dysmenorrhea. In women in which one of these disorders are present, the LNG-IUS has been shown to reduce dysmenorrhea complaints [19, 20]. Therefore, the LNG-IUS still seems to be a good treatment option in women with both HMB and dysmenorrhea. Future research with a special focus on women with combined HMB and dysmenorrhea is necessary to determine which treatment has the highest chance of success.

The presence of menorrhagia as predictor for LNG-IUS failure was investigated in two studies [9, 13]. In both studies it was found that women without severe bleeding had a lower risk of failure. In our study only women with HMB (PBAC-score above 150 points) were included and a higher PBAC-score (≥ 425 points) was not found to be a risk factor for failure. One study investigated if BMI affected the success chance of LNG-IUS treatment and showed the LNG-IUS to be less beneficial in women with a BMI of 25 kg/m^2^ or less [6]. However, this was explained by the greater efficacy of usual medical treatment in leaner women in the control group. Consistent with the results in our study, no significant influence of BMI was found in the LNG-IUS group itself [6]. Two studies investigated the presence of myomas as risk factors for failure, of which one study found women with a myoma of 2.5 cm or larger had a higher risk of failure [10], and the other study found no influence [11]. In our study women with intracavitary or large intramural myomas were excluded because we expected the LNG-IUS and the compared treatment (endometrial ablation) to be less effective in these women. Therefore, we could not investigate the influence of myomas in the present study. In one study an association was found between depression symptoms and receiving a hysterectomy during LNG-IUS treatment (adjusted OR of 3.30) [12]. We did not measure depression symptoms in our study and no other studies were found which showed this association.

Overall, the results of this study can be used to inform women who are below 45 years old and or have severe dysmenorrhea about the higher risk of needing an additional intervention. More research, in a larger study population with specific attention to underlying pathology may identify additional prognostic factors for LNG-IUS failure. In other frequently applied HMB treatments as endometrial ablation, younger age and dysmenorrhea were also found to be risk factors for receiving a reintervention [18]. Therefore, these factors will not be a distinguishing factor to either advise or discourage the use of LNG-IUS treatment. Based upon the available evidence counselling should not be specified to differences in patient characteristics but should instead rather focus on the expectation of women and the specific features of the available treatment options in order for women to make a well-informed decision. Future research comparing differences between prognostic factors for the different treatment options for HMB might be helpful to personalize counselling of women with HMB.

## Conclusion

High discontinuation rates are found in women who receive an LNG-IUS to treat HMB. In this study a younger age and severe dysmenorrhea were found to be risk factors for discontinuation of treatment. These results can be taken into account when counselling women with HMB. However, given the lack of evidence for other possible predictors, patient profiling is not yet helpful to predict success of LNG-IUS treatment, nor to reduce discontinuation rates. Instead counselling should focus on the expectation of women and the differences between the available treatment options, like differences in treatment characteristics and overall effectiveness enabling women to make a well-informed decision.

## Data Availability

The data that support the findings of this study are available from the corresponding author upon reasonable request.

## Abbreviations

CI: Confidence interval
HMB: Heavy menstrual bleeding
LNG-IUS: Levonorgestrel releasing intrauterine system
PBAC: Pictorial blood assessment chart
RR: Relative risk
